# Supplementary material for: Polyphenols Extracted from Shanxi-Aged Vinegar Inhibit Inflammation in LPS-Induced RAW264.7 Macrophages and ICR Mice via the Suppression of MAPK/NF-κB Pathway Activation
Source: Molecules. 2021 May 7;26(9):2745. doi: 10.3390/molecules26092745 (PMC8124351; doi:10.3390/molecules26092745)
Supplement: Supplementary file 1 [file molecules-26-02745-s001.zip › molecules-1196806-supplementary.pdf]

Supplementary Data

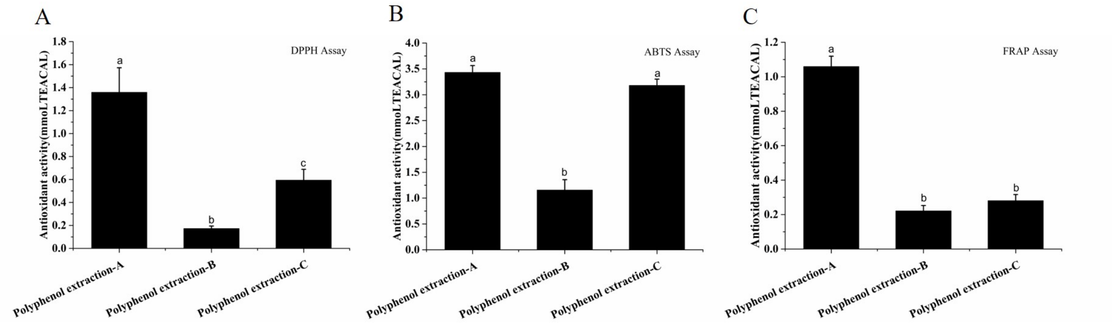

Figure S1. Evaluation of antioxidant activity of polyphenol extracts. (A)DPPH method. (B) ABTS method. (C) FRAP method. Data represent the mean ± SD (n = 6). Values in the same row with different letters are significantly different (P<0.05).

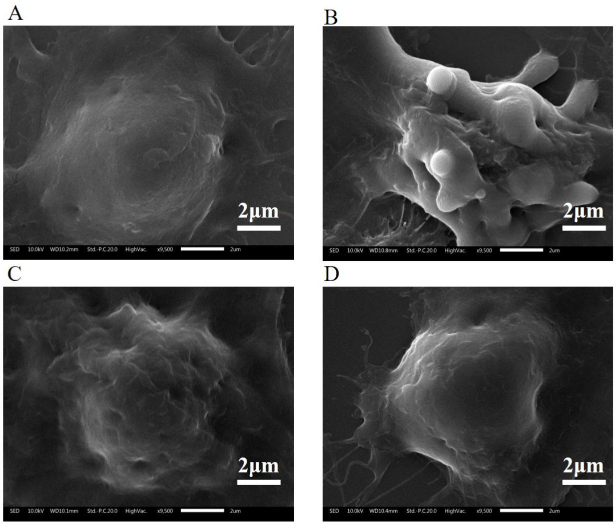

Figure S2. Effects of SAVEP on RAW264.7 cell morphology of LPS-induced inflammatory injury with SEM (9500×). (A) Vehicle group (B) Model group (C) LD group (D) HD group.

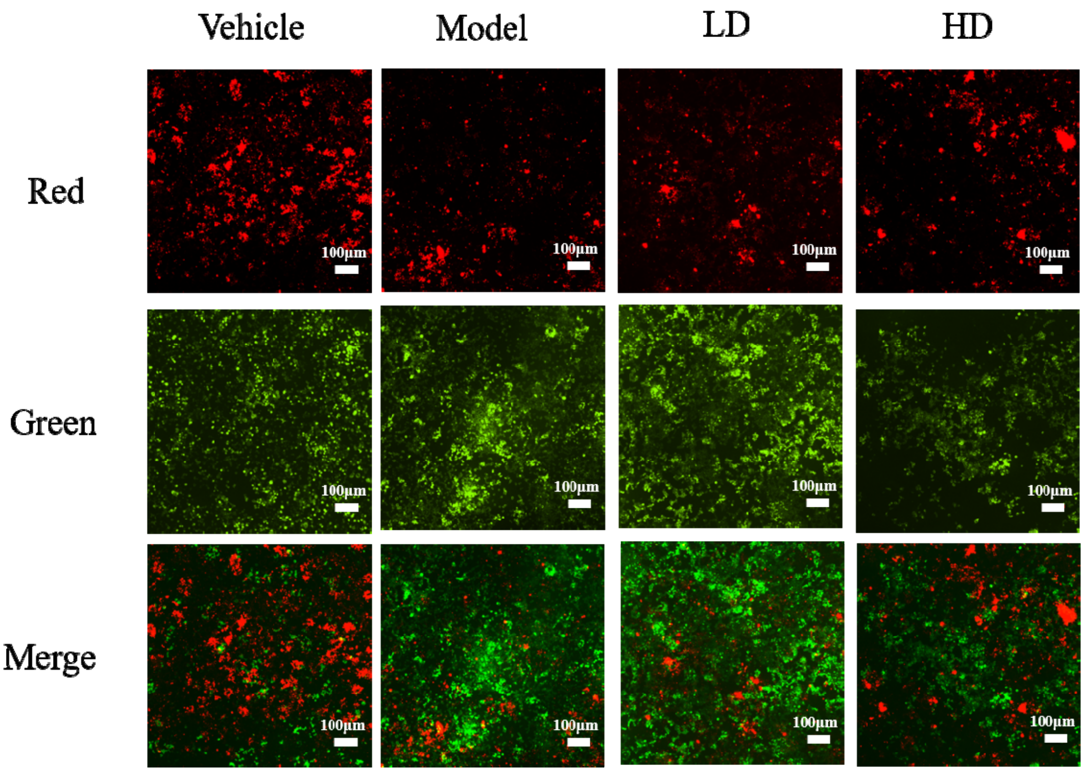

Figure S3. Fluorescence intensity observed by fluorescence microscope. A larger version of Figure 4A

Name is the name of polyphenol derivatives, and Structure is the structure of polyphenol.

Table S1 Results of polyphenol detected by GC-MS in SAVEP

	Name	Retention time (min)	Volume	Concentration (mg/mL)	Structure
Internal Standard Substance	2,4,5-Trihydroxybenzoic acid, 4TMS derivative	28.34±0.34	4320238±51843	0.3000	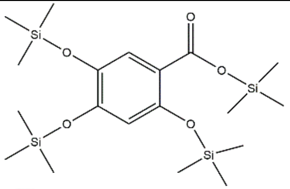
1	Benzaldehyde, 4-[(trimethylsilyl)oxy]-	14.61±0.16	558675±6145	0.0400±0.0004	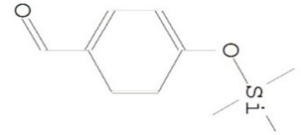
2	2-Hydroxyphenethyl alcohol, di(trimethylsilyl) ether	17.40±0.26	142881±2143	0.0100±0.0002	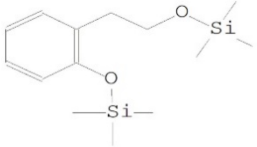
3	Benzaldehyde, 3-methoxy-4-[(trimethylsilyl)oxy]-	18.76±0.38	1210175±24204	0.0800±0.0016	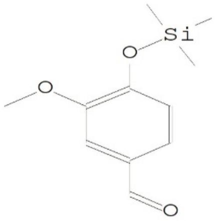
4	Benzoic acid, 4-[(trimethylsilyl)oxy]-, trimethylsilyl ester	21.06±0.21	3049442±30494	0.2100±0.0021	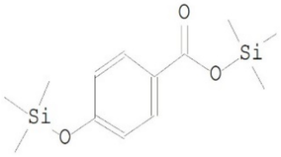

5	Benzeneacetic acid, 4-[(trimethylsilyl)oxy]-, trimethylsilyl ester	21.32±0.23	1117489±12292	0.0800±0.0009	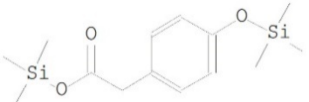
6	Benzeneacetic acid, 3-[(trimethylsilyl)oxy]-, trimethylsilyl ester	22.56±0.43	412186±7832	0.0300±0.0006	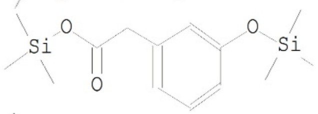
7	Hydrocinnamic acid, p-(trimethylsiloxy)-, trimethylsilyl ester	24.14±0.53	1231460±27092	0.0900±0.0020	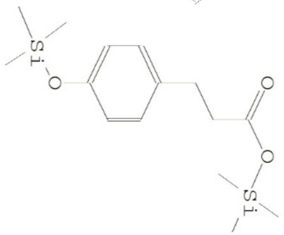
8	Benzoic acid, 3-methoxy-4-[(trimethylsilyl)oxy]-, trimethylsilyl ester	24.22±0.34	5731194±80237	0.4000±0.0056	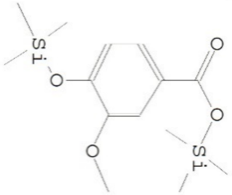
9	Trimethylsilyl [3-methoxy-4-(trimethylsilyloxy)phenyl]acetate	24.34±0.49	655656±13113	0.0500±0.0010	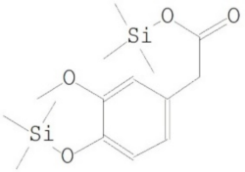
10	Benzoic acid, 3,4-bis[(trimethylsilyl)oxy]-, trimethylsilyl ester	25.45±0.25	2617311±26173	0.1800±0.0018	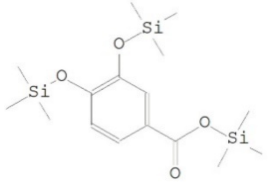

11	Benzenepropanoic acid, 3-methoxy-4[(trimethylsilyl)oxy]-, trimethylsilyl ester	27.04±0.59	6046404±133021	0.4200±0.0092	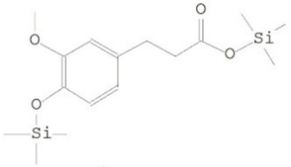
12	Trimethylsilyl 3,5-dimethoxy-4-(trimethylsilyloxy)benzoate	27.11±0.24	1461171±13151	0.1000±0.0009	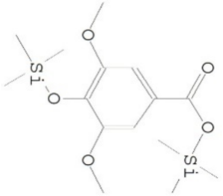
13	Cinnamic acid, p-(trimethylsiloxy)-, trimethylsilyl ester	28.01±0.03	3065278±67436	0.2100±0.0046	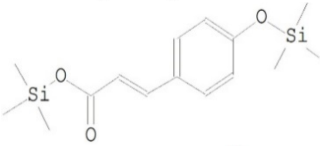
14	Trimethylsilyl 3-methoxy-4-(trimethylsilyloxy)cinnamate	31.06±0.47	1795282±17953	0.3000±0.0030	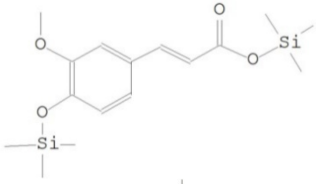
15	(4-Hydroxy-3-methoxyphenyl) ethylene glycol tris(trimethylsilyl) ether	39.27±0.47	1823400±20057	0.1200±0.0013	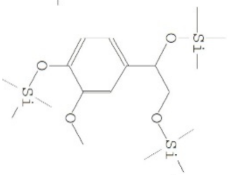
16	(3-Hydroxy-4-methoxyphenyl) ethylene glycol tris(trimethylsilyl) ether	44.24±1.33	1076621±20456	0.1800±0.0034	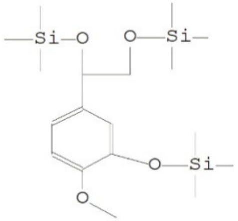

17	(4-Hydroxy-3-methoxyphenyl) ethylene glycol tris(trimethylsilyl) ether	44.52±0.27	942754±20741	0.1300±0.0029	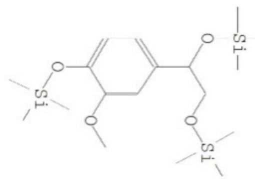
18	Cinnamic acid, p-(trimethylsiloxy)-, trimethylsilyl ester	44.99±0.45	630819±8831	0.0700±0.0010	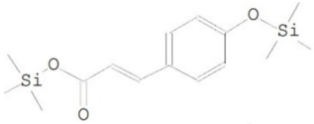
19	(4-Hydroxy-3-methoxyphenyl) ethylene glycol tris(trimethylsilyl) ether	46.19±0.09	480837±9617	0.0700±0.0014	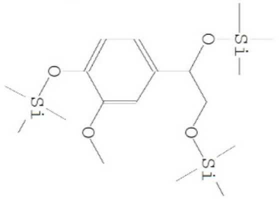

Date in the table are represent mean ± SD (n = 3)
